# Advancing Biomechanics-Based Motion Analysis from Methodology to Application

**DOI:** 10.3390/bioengineering12111200

**Published:** 2025-11-03

**Authors:** Chen He, Hong Fu, Christina Zong-Hao Ma

**Affiliations:** 1 School of Health Science and Engineering, University of Shanghai for Science and Technology, Shanghai 200093, China; hechen@usst.edu.cn; 2 Department of Mathematics and Information Technology, The Education University of Hong Kong, Hong Kong SAR 999077, China; hfu@eduhk.hk; 3 Department of Biomedical Engineering, The Hong Kong Polytechnic University, Hong Kong SAR 999077, China; 4 Research Institute for Smart Ageing, The Hong Kong Polytechnic University, Hong Kong SAR 999077, China

## 1. Introduction

Biomechanics-based motion analysis provides the foundation for understanding human movement, from diagnosing pathological gait to optimizing athletic performance. The field is now evolving from traditional subjective observation to objective data-driven analysis, through advanced technologies in real-world environments. This special issue compiles ten original research articles that not only refine measurement methodologies but also demonstrate their significant impact on addressing clinical and sports-related challenges. The contributions examine two complementary areas: advanced methodology development and their practical implementation. Through technologies such as deep learning, wearable sensors, and computational modeling, these studies collectively enhance the precision, accessibility, and ecological validity of motion analysis across diverse movement contexts.

## 2. Contributions to This Special Issue

### 2.1. Methodological Innovations in Motion Analysis

A prominent focus in motion analysis is the continuous improvement of the tools and techniques for data acquisition and analysis, which prioritizes accuracy, accessibility, and applicability. Several studies in this Special Issue showcase significant progress in measurement technologies and computational modeling. Torvinen et al. [1] validated a deep learning-based 3D markerless motion capture system for skate skiing, demonstrating high agreement with marker-based systems. This system provides a promising tool for performance analysis and technique monitoring in sports. Similarly, Buckley et al. [2] validated the wearable Inertial Measurement Units (IMUs) against the clinical standard of overnight videography for assessing sleep biomechanics, confirming their reliability for efficient, home-based sleep monitoring.

Furthermore, Del Vecchio et al. [3] identified and mitigated movement artifacts in chronic pallidal local field potential recordings using IMU regression, which is a critical step to ensure the fidelity of neural biomarkers in adaptive deep brain stimulation. In parallel, Liang et al. [4] utilized a finite element model incorporating Hill-type muscle dynamics to simulate forearm flexion. Their simulations identified reduced fast-twitch fiber percentage, muscle strength, and neural excitation as key factors to diminished forearm mobility in the aging population, providing a computational framework to diagnose movement disorders.

### 2.2. Applications in Clinical and Sports Science

The ultimate value of biomechanical analysis lies in its application to address clinical and performance challenges. Some studies provide critical insights into pathophysiology, rehabilitation, and injury prevention through motion analysis. Ablove et al. [5] demonstrated that standing position produces the lowest pelvic floor pressures, with peak pressure localized at the symphysis. These biomechanical patterns explain both the protective nature of upright posture and the predisposition for anterior compartment prolapse. Meanwhile, Triantafyllou et al. [6] reported that IMU-based kinematic analysis during lumbar bridge tests were different in patients with chronic low back pain and healthy controls, which provides quantifiable diagnostics for evaluating rehabilitation efficacy. Further advancing neurorehabilitation, Joshi et al. [7] illustrated the potential of targeted neuromodulation, showing that trunk-specific spinal cord epidural stimulation can restore upright postural control in individuals with spinal cord injury.

In sports science, Zhang et al. [8] identified lower leg and knee stiffness in female athletes during a badminton landing task. The reduced dynamic stiffness indicated diminished joint stability and presented a biomechanical mechanism for the possible gender-related differences in non-contact anterior cruciate ligament injuries. Complementing this, Tong et al. [9] investigated the neuromuscular responses during standing balance challenges, and found that agonist muscles, particularly ankle muscles, activated fastest to counter perturbations, with muscle reaction speeds scaling with perturbation intensity. This hierarchy of control provides valuable insights for developing evidence-based fall-prevention protocols. Shim et al. [10] examined motor control in the absence of visual feedback, and demonstrated the remarkable capacity of the proprioceptive system to maintain bilateral symmetry and execute precise reaching movements under elastic loads, providing important implications for visual-deprived rehabilitation protocols.

## 3. Conclusions

This Special Issue presents a collection of studies that bridge advanced measurement technologies with critical applications in rehabilitation and sports science. These developments point toward increasingly personalized, data-driven approaches that translate biomechanical insights into optimized rehabilitation protocols and enhanced performance outcomes, thereby advancing both human health and athletic achievement.
